# AIM interneurons mediate feeding suppression through the TYRA-2 receptor in *C. elegans*

**DOI:** 10.1007/s41048-018-0046-2

**Published:** 2018-03-05

**Authors:** Jiajun Fu, Haining Zhang, Wenming Huang, Xinyu Zhu, Yi Sheng, Eli Song, Tao Xu

**Affiliations:** 1 National Laboratory of Biomacromolecules, CAS Center for Excellence in Biomacromolecules, Institute of Biophysics, Chinese Academy of Sciences, Beijing, 100101 China; 2 College of Life Sciences, University of Chinese Academy of Sciences, Beijing, 100049 China; 3 Key Laboratory of Molecular Biophysics of the Ministry of Education, College of Life Science and Technology, Huazhong University of Science and Technology, Wuhan, 430074 China

**Keywords:** *C. elegans*, TYRA-2 receptor, AIM interneurons, Peripheral feeding regulation, Tyramine

## Abstract

Feeding behavior is the most fundamental behavior in *C. elegans*. Our previous results have dissected the central integration circuit for the regulation of feeding, which integrates opposing sensory inputs and regulates feeding behavior in a nonlinear manner. However, the peripheral integration that acts downstream of the central integration circuit to modulate feeding remains largely unknown. Here, we find that a Gαi/o-coupled tyramine receptor, TYRA-2, is involved in peripheral feeding suppression. TYRA-2 suppresses feeding behavior via the AIM interneurons, which receive tyramine/octopamine signals from RIM/RIC neurons in the central integration circuit. Our results reveal previously unidentified roles for the receptor TYRA-2 and the AIM interneurons in feeding regulation, providing a further understanding of how biogenic amines tyramine and octopamine regulate feeding behavior.

## Introduction

Animals face complex variation in their living environments and must alter their behaviors in response to internal and/or external environmental changes. The neural circuits and signal transmission pathways underlying these behavioral changes can be examined in detail in the nematode *C. elegans*, since all neurons and their entire connectivity patterns have been described (White *et al.* 1986). *C. elegans* is equipped with multiple sensory modalities that can detect various environmental cues, including odors, tastes, osmolarity, temperature, and mechanical touch. It can sense hundreds of water-soluble and volatile molecules, which can evoke distinct behaviors, such as attraction, avoidance, mating, or feeding (Bargmann 2006; Bergamasco and Bazzicalupo 2006).

In *C. elegans*, the feeding state is dynamically regulated by several environmental cues, including odors, tastes, temperature, and nutrient states (Avery and Horvitz 1990; Jones and Candido 1999; Li *et al.* 2012). However, the neural integration processes underlying the regulation of feeding are not well defined. Our previous research has dissected a central “flip-flop” circuit in feeding regulation (Li *et al.* 2012). In that model, attractive odors sensed by the AWA neuron will activate the serotonergic neuron NSM to release serotonin, thereby ultimately increasing the feeding rate. Meanwhile, bad tastes sensed by ASH will activate the interneurons RIM and RIC, which release tyramine and/or octopamine to suppress feeding. Specifically, the two kinds of biogenic amines inhibit each other’s function: serotonin inhibits the activation of RIM and RIC neurons via the MOD-1 receptor and tyramine/octopamine inhibits NSM neurons via the SER-2 receptor. This central cross-inhibition integration circuit ensures a nonlinear, bistable output.

Besides the central integration circuit, neural regulatory mechanisms at the peripheral sites remain largely elusive. Here, by integrating calcium imaging, optogenetic interrogation, genetic manipulation, and behavioral analysis, we present evidence that TYRA-2, a Gαi/o-coupled tyramine receptor, which acts as a signal target at the peripheral site in the AIM interneurons, receives signals from RIM/RIC neurons in the central integration circuit to suppress feeding behavior. *tyra*-*2* mutant worms show defects in the suppression of feeding by 1-octanol, whereas overexpression of TYRA-2 under its own promoter or the *zig*-*3* promoter, which is specifically expressed in the AIM interneurons, can rescue the feeding suppression defect. Furthermore, calcium imaging data show that TYRA-2 functions in the modulation of feeding by inhibiting the AIM interneurons, and optogenetic silencing of AIM neurons mimics that feeding suppression.

## Results

### TYRA-2 is required for feeding suppression by 1-octanol

**Fig. 1:**
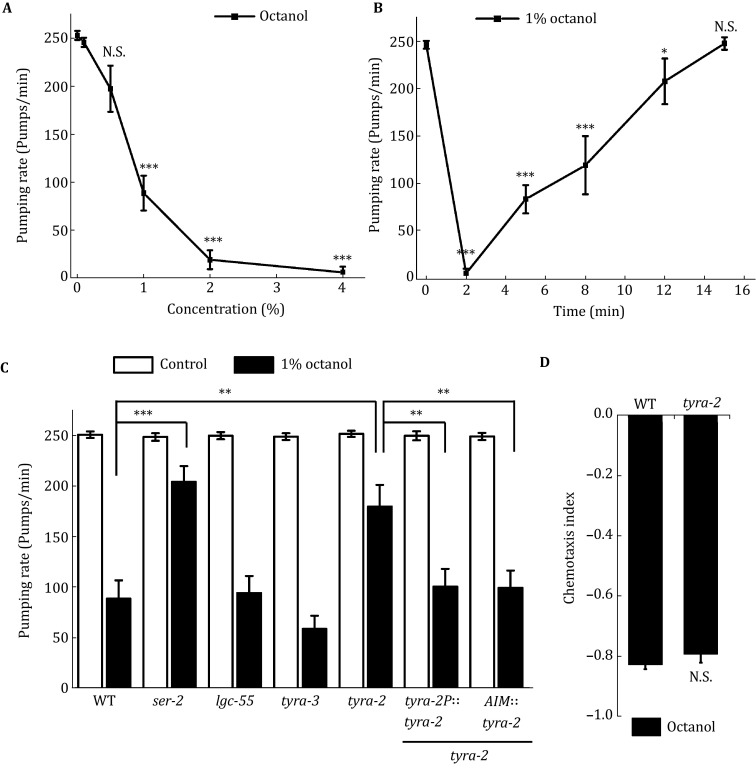
TYRA-2 is required for feeding suppression by 1-octanol. **A** *C. elegans* pumping rate in response to various concentrations of diluted 1-octanol. **B** 1-octanol at 1% inhibits *C. elegans* feeding in a time-dependent manner. **C** Feeding suppression induced by 1% 1-octanol shows a significant defect in *ser*-*2* or *tyra*-*2* mutants. Exogenous expression of TYRA-2 under its own promoter or specifically in AIM interneurons could fully rescue its feeding suppression defect in the *tyra*-*2* mutant. **D** *tyra*-*2* mutant worms show a chemotaxis index comparable to that of wild-type worms. *p < 0.05, **p < 0.01, ***p < 0.001. “N.S.” indicates no significant difference

1-Octanol is a volatile molecule that is known as a repellent to evoke avoidance behavior in *C. elegans* (Bargmann *et al.* 1993; Chao *et al.* 2004). In our study, we found that in addition to inducing avoidance behavior, 1-octanol also inhibited feeding in a time- and dose-dependent manner (Fig. 1A, B). 1-octanol at concentrations of 1% or higher significantly suppressed *C. elegans* pharyngeal pumping, and that suppression was sustained for more than 10 min, providing a suitable model for studying the neural circuits in feeding suppression.

By employing 1-octanol in the feeding suppression assay, we tried to identify the neurons and receptors downstream of tyramine, which acts as a crucial transmitter in the process of feeding suppression (Li *et al.* 2012). There are four tyramine receptors in *C. elegans*, including three G protein-coupled tyramine receptors, TYRA-2, TYRA-3, SER-2, and a tyramine-gated chloride channel LGC-55 (Rex *et al.* 2005; Rex and Komuniecki 2002; Ringstad *et al.* 2009). Our behavioral experiments showed that feeding inhibition evoked by 1% 1-octanol had a dramatic defect in the *ser*-*2* mutant, which is involved in central integration of feeding (Li *et al.* 2012). Another mutant, *tyra*-*2,* also showed a significant defect compared with wild-type worms (Fig. 1C).

Exogenous expression of TYRA-2 under its own promoter could fully rescue its feeding suppression defect (Fig. 1C). We observed that the *tyra*-*2* mutant worms showed a 1-octanol chemotaxis index similar to that of wild-type worms (Fig. 1D), so the difference might result from a cause other than altered 1-octanol avoidance behavior. These data suggest that TYRA-2 is required for the peripheral regulation of feeding suppression.

### TYRA-2 acts in the AIM interneurons to modulate feeding suppression

**Fig. 2:**
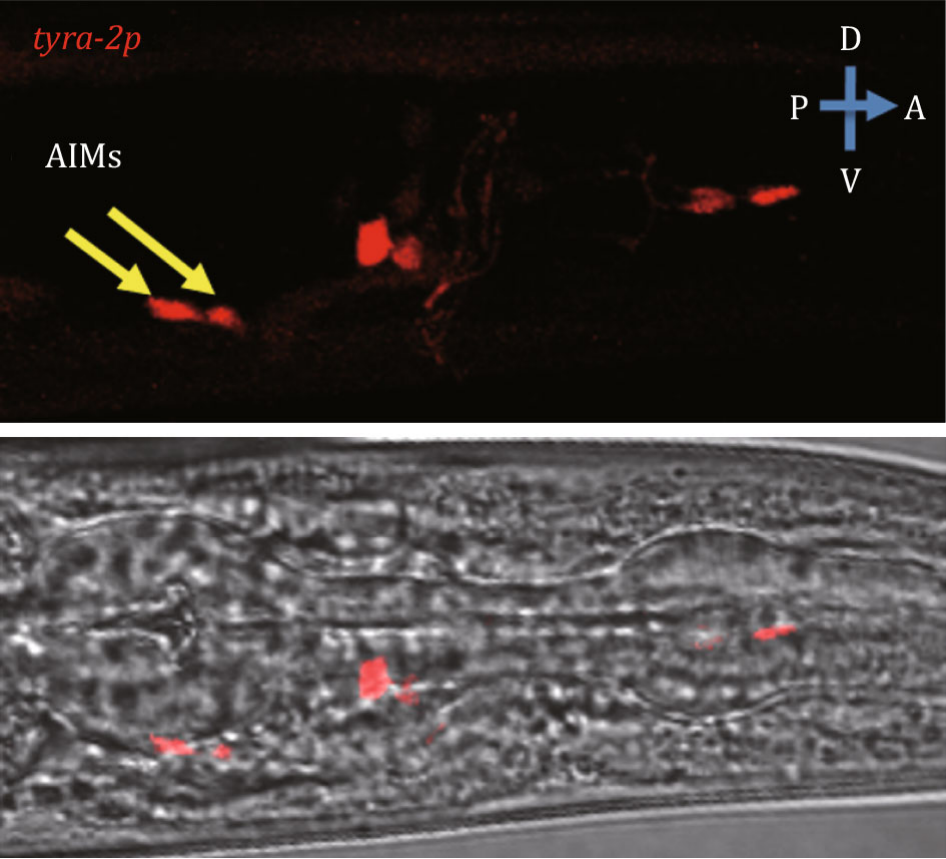
The expression of TYRA-2 in AIMs. The plasmid *tyra*-*2p*::RFP was constructed to identify the expression pattern of TYRA-2. Pictures were captured by laser scanning confocal microscopy. Animal was shown with anterior to the right. *Yellow arrows* indicate AIMs

In *C. elegans*, TYRA-2 is expressed exclusively in neurons, including the sensory neurons ASH, ASE, ASG, and ASI, the body and tail neurons AVM, ALM, PVM, and PLM, and the AIM interneurons (Fig. 2) (Rex *et al.* 2005). Considering the function of TYRA-2 in the peripheral integration of feeding suppression, the AIM interneurons become the most likely candidate. Expression of TYRA-2 in AIMs by the *zig*-*3* promoter, which is reported to be specifically expressed in AIM neurons (Altun-Gultekin *et al.* 2001; Aurelio *et al.* 2002), was sufficient to rescue the feeding suppression defect in *tyra*-*2* mutant worms (Fig. 1C). These experiments indicate that TYRA-2 acts in AIM neurons.

### TYRA-2 activation results in the silencing of AIMs

**Fig. 3:**
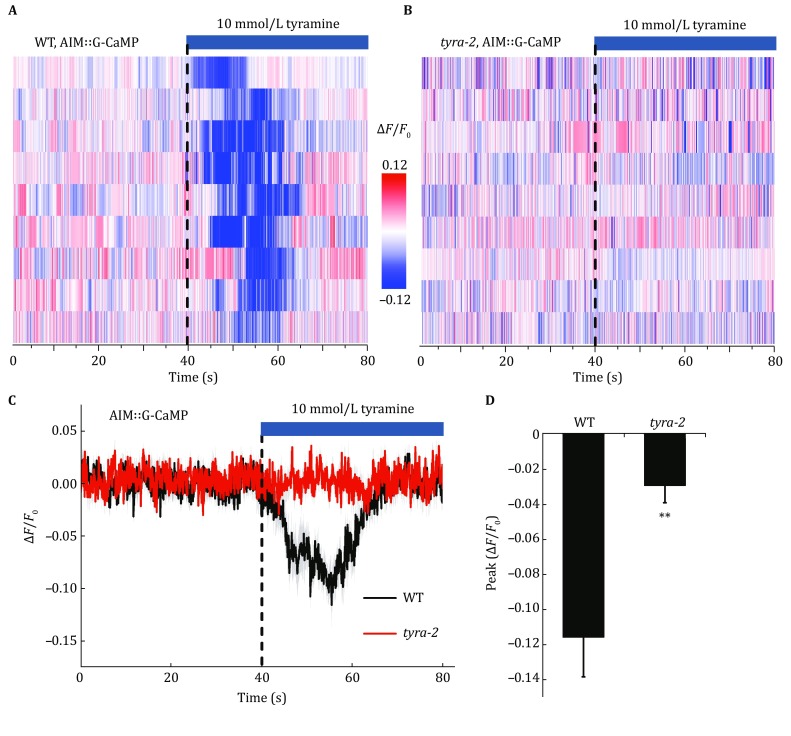
Tyramine inhibits AIM interneurons via TYRA-2 receptor**. A**, **B** Stimulation with 10 mmol/L tyramine inhibits AIM interneurons in wild-type worms but not in *tyra*-*2* mutants. The calcium indicator GCaMP3 was used to monitor the calcium transient in AIM neurons. *Dashed lines* indicate the time of tyramine application. **C**, **D** The average lines (**C**) and peak amplitudes (**D**) of AIM calcium transients in response to tyramine stimulation in wild-type and *tyra*-*2* mutant worms. The shades around the average traces indicate error bars (SEM). **p < 0.01

TYRA-2 encodes a Gα_i/o_-coupled tyramine receptor with high affinity with a *K*_d_ of 20 ± 5 nmol/L (Rex *et al.* 2005). To test the effect of TYRA-2 on AIM, we monitored the calcium transients of AIM interneurons upon tyramine stimulation by specifically expressing GCaMP3 in AIM. It turned out that apart from the dramatic suppression of the feeding rate, tyramine also reduced the calcium level in AIM neurons (Fig. 3A, C, D). Whereas, *tyra*-*2* mutation blocked the AIMs calcium reduction in response to tyramine (Fig. 3B, C, D). These results indicate that AIM neurons are silenced during the tyramine-mediated feeding suppression via the receptor TYRA-2.

### Silencing of AIM interneurons induces feeding suppression

**Fig. 4:**
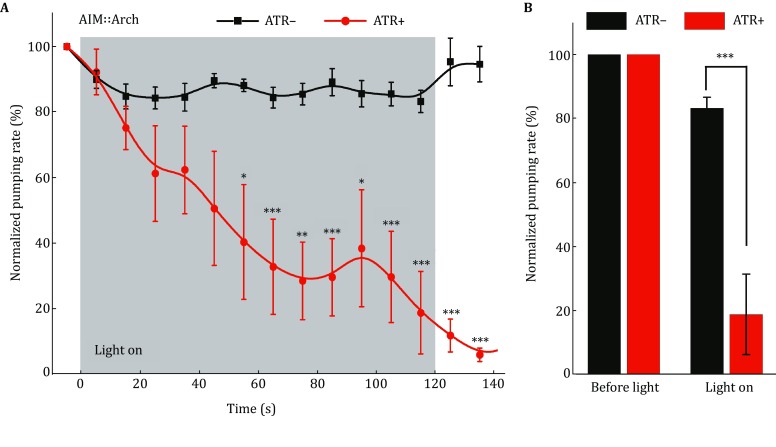
Optogenetic silencing of AIM interneurons mimics the feeding suppression. **A** Optogenetic silencing of AIM interneurons by green light (550 nm, 1.77 mW/mm^2^) suppresses *C. elegans* pharyngeal pumping in a time-dependent manner. **B** The pumping rates before and after optogenetic stimulation. Worms expressing Arch in AIMs were tested and the transgenic animals cultured on ATR-free plates were used as controls. *p < 0.05, **p < 0.01, ***p < 0.001

If AIM interneurons indeed regulate feeding suppression as suggested by behavioral and calcium imaging experiments, acute silencing of AIM should affect the pumping rate. To test this, we took an optogenetic approach by expressing the light-driven outward proton pumps archaerhodopsins (Arch) (Chow *et al.* 2012) specifically in AIM interneurons using the *zig*-*3* promoter. Archaerhodopsins require the cofactor all-trans-retinal (ATR) as their essential chromophore, which acts to capture light (Chow *et al.* 2012). In this optogenetic manipulation, worms cultured without all-trans-retinal were used as controls. As predicted, we found that compared with control worms, pumping behavior was dramatically slowed by the optogenetic silencing of AIM interneurons (Fig. 4). These data confirm that silencing of AIM by TYRA-2 would mediate feeding suppression.

## Discussion

**Fig. 5:**
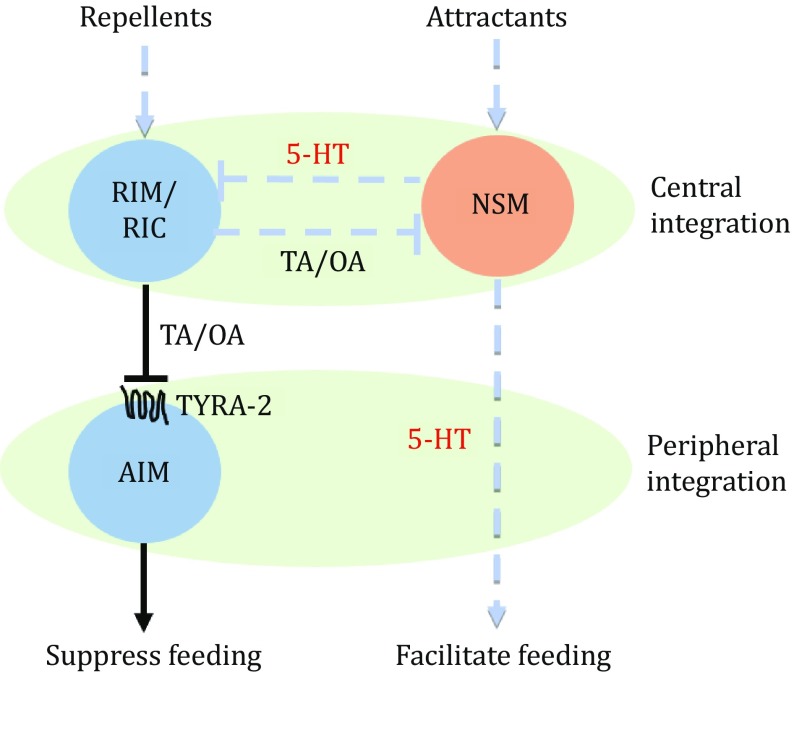
A schematic model of the feeding regulation circuit. Serotonin and tyramine/octopamine function as crucial neural transmitters in the central integration circuit of the feeding regulation, while the receptor TYRA-2 and the interneuron AIM function in the peripheral integration circuit downstream of tyramine/octopamine. 5-HT: serotonin, TA: tyramine, OA: octopamine

In our previous reports, we have identified tyramine as an important transmitter for feeding suppression and shown that SER-2, a tyramine receptor, is involved in the central “flip-flop circuit” (Li *et al.* 2012). Apart from the central integration circuit, here, we identified for the first time that TYRA-2 function cell autonomously in the AIM interneuron was involved in the peripheral feeding regulation. Tyramine released from RIM/RIC neurons would inhibit AIM by the Gα_i/o_-coupled receptor TYRA-2, resulting in feeding suppression (Fig. 5).

AIM interneurons are reported to mediate swim initiation by serotonin signaling (Vidal-Gadea *et al.* 2011) and mate-searching behavior of males by PDF-1 neuropeptide signaling (Barrios *et al.* 2012). In contrast, little is known about their functions in feeding regulation. Here, we identified that AIM interneurons were inhibited by the crucial feeding modulator tyramine, and that resulted in feeding suppression. The tyraminergic neurons RIM/RIC share no direct synaptic connections with AIM neurons. But given the fact that these neurons localized very close to each other, it is possible that the signal transmission may be mediated by extrasynaptic tyramine receptors on AIM neurons. AIM interneurons are equipped with two kinds of classical neurotransmitters, glutamate and serotonin, as well as several neuropeptides (Li and Kim 2008; Serrano-Saiz *et al.* 2013; Sze *et al.* 2000). Among them, serotonin is known as a crucial neuromodulator that facilitates feeding (Avery and Horvitz 1990; Sze *et al.* 2000). The inhibition of AIM may block the serotonin release and result in slower feeding behavior.

In *C. elegans*, tyramine is the precursor of octopamine (Alkema *et al.* 2005). Tyramine is formed by the decarboxylation of tyrosine, dependent on a tyrosine decarboxylase gene, *tdc*-*1*. Meanwhile, octopamine is formed by the hydroxylation of tyramine, which depends on a tyramine β-hydroxylase gene, *tbh*-*1 (*Alkema *et al.* 2005*)*. It has been reported that tyramine and octopamine may function independently in the modulation of egg laying, as well as reversal behavior and head oscillation suppression in response to anterior touch (Alkema *et al.* 2005). However, they seem to inhibit pumping in the same way (Greer *et al.* 2008; Li *et al.* 2012). In fact, in addition to its high affinity to tyramine, TYRA-2 also has a relatively high affinity to octopamine (Rex *et al.* 2005). Thus, the TYRA-2-induced AIM inhibition may be caused by tyramine and/or octopamine from RIM/RIC neurons.

*tyra*-*2* encodes a Gαi/o protein-coupled tyramine receptor (Rex *et al.* 2005). In *C. elegans*, Gαi/o subunits communicate signals from a series of hormones and neurotransmitters, including serotonin (Mendel *et al.* 1995; Segalat *et al.* 1995), acetylcholine (Bany *et al.* 2003), dopamine (Sawin *et al.* 2000), and FRMFamides (Nelson *et al.* 1998; Rogers *et al.* 2001). *goa*-*1* encodes the only clear member of the mammalian Gαi/o class of Gα subunits, and it is involved in many types of behavior regulations, including locomotion, egg laying, and male mating (Mendel *et al.* 1995; Segalat *et al.* 1995). Moreover, octopamine has been reported to inhibit pumping through the GOA-1 pathway (Keane and Avery 2003). While, how Gαi/o subunits participate in regulating feeding is not clearly defined. Here, we found that the Gαi/o-coupled tyramine receptor TYRA-2 was involved in peripheral feeding modulation.

Tyramine and octopamine are important amines not only in *C. elegans* but also in insects, rats, and human beings, and they are involved in neural circuits, metabolism, and also diseases such as cancer and Parkinson’s diseases (Chen and Wilkinson 2012; Li *et al.* 2012; Rumore *et al.* 2010; Xu *et al.* 2013). However, the functions of these amines in neural circuits remain inconclusive, even simply in *C. elegans*. Here, we found that these amines may suppress feeding via TYRA-2 receptor in AIM interneurons in the peripheral feeding suppression. Our findings provide a novel insight into feeding regulation in *C. elegans* and into the function process of tyramine and octopamine in neuron circuits.

## Materials and methods

### General methods and strains

*C. elegans* were cultured on nematode growth medium (NGM) plates with *E. coli* OP50 in standard procedures (Brenner 1974), and the following strains were used in this study: N2, TM1846: *tyra*-*2(tm1846),* VC125: *tyra*-*3(ok325),* OH313: *ser*-*2(pk1357),* TM2913: *lgc*-*55(tm2913)*, TXL210: *tyra*-*2(tm1846)*; *txuEx210*[*tyra*-*2p::tyra*-*2*::GFP, *lin*-*44p*::GFP], TXL211: *tyra*-*2(tm1846)*; *txuEx211*[*zig*-*3p::tyra*-*2*::GFP, *lin*-*44p*::GFP], TXL212: *txuEx212*[*tyra*-*2p*::RFP, *lin*-*44p*::GFP], TXL213: *lite*-*1(ce314)*; *txuEx213*[*zig*-*3p*::GCaMP3, *zig*-*3p*::mKate2, *lin*-*44p*::GFP], TXL214: *lite*-*1(ce314);tyra*-*2(tm1846)*; *txuEx213*[*zig*-*3p*::GCaMP3, *zig*-*3p*::mKate2, *lin*-*44p*::GFP], TXL215: *lite*-*1(ce314)*; *txuEx215*[*zig*-*3p*::Arch::GFP, *lin*-*44p*::GFP].

### Molecular biology

For *tyra*-*2* rescue experiments, the full-length *tyra*-*2* gene was amplified from the N2 genome. The *tyra*-*2* promoter used in this study was amplified approximately 3 kb upstream from the start codon. For AIM-specific expression, a 4.45 kb promoter of *zig*-*3* was used.

### Behavioral assays

Feeding behavior was assayed as previously described (Li *et al.* 2012). In brief, the pumping rate was calculated by measuring the time required to complete 20 pumps. To test the effect of the volatile chemical 1-octanol, well-fed worms were transferred to new 3-cm NGM plates seeded with OP50. After 10 min, 2 μl various concentrations of 1-octanol (dissolved in 100% ethanol, *vol*/*vol*) were added to the lids before the plates were sealed. The pumping rates were recorded 5 min later, except for the time-dependence curve (Fig. 1B). Three measurements were recorded for each worm, and more than ten worms were tested per experiment.

Chemotaxis assays were performed using the standard protocol as described previously (Yoshida *et al.* 2012). In general, 1 μl of 1 mol/L sodium azide was spotted onto two points at opposite sides of a 10-cm unseeded NGM plate, with 2 μl volatile chemical 1-octanol near the alternative one. Between 100 and 200 well-fed worms were placed near the center of the plate. After 1 h, the numbers of animals near and opposite the 1-octanol, termed N^+^ and N^−^, respectively, were counted. Chemotaxis index = [(N^+^) − (N^−^)]/[(N^+^) + (N^−^)].

All behavioral assays used young adult worms that were cultured and tested at 20 °C. All experiments were repeated at least three independent times.

### Calcium imaging and optogenetics

Calcium imaging was performed on free-moving worms by the imaging system as previously described (Li *et al.* 2012). We measured the calcium activity by neuron-specific expression of the green fluorescent calcium indicator GCaMP3, with the red fluorescent protein mKate2 used as a reference. For optogenetic interrogation, the light-driven outward proton pumps archaerhodopsins were specifically expressed in AIM neurons. Green light (550 nm, 1.77 mW/mm^2^) was presented for 2 min. Worms cultured without all-trans-retinal (ATR) were used as controls. To eliminate the intrinsic photophobic response, all calcium imaging and optogenetic experiments were performed on a *lite*-*1(ce314)* genetic background. At least nine worms were tested for each experiment.

### Statistical analysis

Data analysis is conducted using Origin Pro_9.0.0. Results are presented as the mean ± SEM, and the statistical significance of differences is assessed using the two-tailed *t* test. *p < 0.05. **p < 0.01. ***p < 0.001. N.S. indicates no significant difference.
